# An Interesting Case of Immunoglobulin G4-Related Retroperitoneal Fibrosis Treated With Rituximab

**DOI:** 10.7759/cureus.17940

**Published:** 2021-09-13

**Authors:** Ahmad Hamdan, Zunera Moeen, Hina Tariq, Olga Olson, Carlos Matute-Martinez, Mandeep Sidhu, Srikanth Mukkera

**Affiliations:** 1 Internal Medicine, Texas Tech University Health Sciences Center School of Medicine at the Permian Basin, Odessa, USA; 2 Internal Medicine, Texas Tech University-Permian Basin, Odessa, USA; 3 Internal Medicine, Islam Medical College, Sialkot, PAK; 4 Internal Medicine, Twin Cities Community Hospital, Templeton, USA; 5 Internal Medicine, Texas Tech University Health Sciences Center, Odessa, USA; 6 Rheumatology, Texas Tech University Health Sciences Center, Odessa, USA

**Keywords:** igg4 disease, retroperitoneal fibrosis, rituximab, diagnosis, glucocorticoids

## Abstract

Immunoglobulin G4-related disease (IgG4-RD) is a fibroinflammatory condition. Its common manifestations include type I autoimmune pancreatitis and retroperitoneal fibrosis (RPF). We present a rare case involving a 43-year-old female who presented with left lower quadrant (LLQ) pain. Imaging of the abdomen and pelvis revealed left hydroureteronephrosis to the level of an inflammatory process in the left adnexal region, possibly reflecting a tubo-ovarian abscess (TOA). The gynecologic evaluation concluded that the mass was unlikely of gynecologic sources. Transgluteal biopsy of the mass was highly suggestive of IgG4-RD. The patient received prednisone and rituximab (RTX), resulting in complete resolution of the mass, which was confirmed on repeat imaging. This case report provides a valuable addition to the literature to highlight that the diagnosis of IgG4-RD is based on the combination of characteristic clinical, serologic, radiologic, and histopathologic findings. Also, it underlines that the management of the disease is through glucocorticoids (GCs) as the first-line agent for remission induction in all patients with active, untreated IgG4-RD. RTX therapy is an effective treatment for IgG4-RD that is refractory to GCs. Recent studies have suggested that RTX monotherapy can be used to induce and maintain remission in patients with IgG4-RD.

## Introduction

Immunoglobulin G4-related disease (IgG4-RD) is an immune-mediated fibroinflammatory condition that can affect multiple organs. Its common manifestations include type I autoimmune pancreatitis, IgG4-related sclerosing cholangitis, and retroperitoneal fibrosis (RPF). Retroperitoneal involvement is reported in 20-56% of patients with IgG4-RD, and IgG4-RD is responsible for 57-59% of idiopathic RPF [1].

RPF develops insidiously as the initial symptoms are nonspecific. Most common symptoms include low-back, flank, or abdominal pain, often radiating to the groin and/or side of the thigh. RPF related to IgG4-RD shows unilateral hydronephrosis in 75% of cases [2].

Diagnosis of IgG4-RD is based on the combination of characteristic clinical, serologic, radiologic, and histopathologic findings. Glucocorticoids (GCs) are the first-line agents for remission induction in all patients with active IgG4-RD [3]. In select patients, the combination of GCs and a steroid-sparing immunosuppressive agent may be beneficial as the initial treatment. Rituximab (RTX) is a CD20 monoclonal antibody and B-cell-depleting agent and is an effective management option for patients who are refractory to GCs and other medications [4].

## Case presentation

A 43-year-old female with a past medical history of uncontrolled type II diabetes mellitus, recurrent urinary tract infections (UTIs), chronic pelvic inflammatory disease (PID), abnormal colposcopy with the highest grade cervical intraepithelial neoplasia (CIN) II status post-cryotherapy, and bilateral tubal ligation (BTL) presented with worsening left lower quadrant (LLQ) pain of two months' duration. On review of systems, the patient endorsed yellow/green vaginal discharge of two months' duration without a malodor or pruritus.

The remainder of the review was negative, particularly for other gynecologic, urinary, and rheumatologic symptoms including but not limited to fever, chills, weight loss, rash, Raynaud’s skin changes, photophobia, blurred vision, dry eyes or mouth, oral ulcers, joint pain, swelling or redness, dysuria, hematuria, or urinary urgency or incontinence. The patient denied any history of autoimmune disease in herself or her family. Her only home medication was metformin. Vitals were within normal limits. Abdominal examination was significant for mild LLQ tenderness without rebound or guarding. On pelvic examination, external genitalia was normal; speculum exam revealed a well-rugated vagina with thick green discharge on the vaginal walls and cervical epithelium and a friable cervix. Pap smear was performed and was negative for intraepithelial lesions or malignancy. Bimanual examination revealed an irregular, hard, smooth, nonmobile, and tender 5-cm mass on the left anterior vaginal wall starting from the left side of the cervix and extending to the pelvic sidewall that is not connected to the cervix. No cervical motion tenderness was noted. The uterus was mobile, anteverted, and non-tender with a smooth contour. Adnexa was tender bilaterally but nonpalpable. The mass was also palpable with similar characterization, as observed through a digital rectal examination.

Transabdominal and transvaginal ultrasound (U/S) showed a complex left adnexal mass that could represent a left hydrosalpinx and/or tubo-ovarian abscess (TOA), left hydroureteronephrosis with normal-appearing right adnexa, and uterus with an endometrial stripe of 0.9 cm. A CT scan of the abdomen and pelvis revealed left hydroureteronephrosis to the level of a complex inflammatory process in the left adnexal region, possibly reflecting an evolving TOA with a trace amount of gas in the bladder and mild bladder wall thickening and perivesical fat stranding.

Labs were significant for leukocytosis of 11.2. Urinalysis (UA) was negative for leukocyte esterase, nitrites, and white and red blood cells. The wet mount was negative. Alpha-fetoprotein (AFP), carcinoembryonic antigen (CEA), cancer antigen (CA) 125, B-human chorionic gonadotropin (B-HCG), chlamydia, gonorrhea, human papillomavirus (HPV), hepatitis B, hepatitis C, and HIV tests were negative. Autoimmune workup with antinuclear antibody (ANA), rheumatoid factor (RF), and anticitrullinated peptide (anti-CCP) were also negative. Blood and urine cultures returned negative as well.

**Figure 1:**
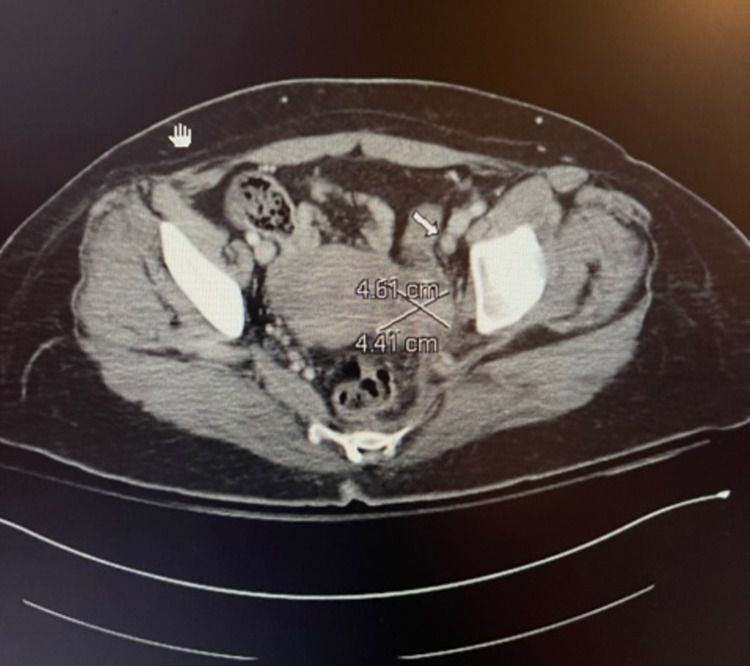
CT abdomen and pelvis on admission Left adnexa mass is 4.61 x 4.41 cm in dimension; left side ureter is compressed by the mass posteriorly (white arrow) CT: computed tomography

Given the patient's history of PID and abnormal CIN II, we consulted gynecology. They performed endocervical curettage and endometrial biopsy, which were negative for cytologic atypia or malignancy. They later performed posterior colpotomy, cystoscopy, and diagnostic laparoscopy with peritoneal biopsy, which revealed normal pelvic anatomy with healthy ovaries, surgical absence of fallopian tubes consistent with the history of BTL, and an irregular solid hard mass in the left retroperitoneal space, not communicating with the uterus or the cervix, not visualized to be impinging on the bladder, and not visible from the abdomen on diagnostic laparoscopy. Attempts to biopsy the mass under U/S guidance were unsuccessful. They concluded the mass to be unlikely from gynecological sources. Later, an abdominal and pelvic MRI was obtained, which demonstrated a retroperitoneal mass with increased enhancement adjacent to the left ovary. A CT urogram was also done, which showed a nonspecific 4.61 x 4.41-cm soft tissue density within the left adnexa that could represent TOA or a neoplasm (Figure 1).

**Figure 2:**
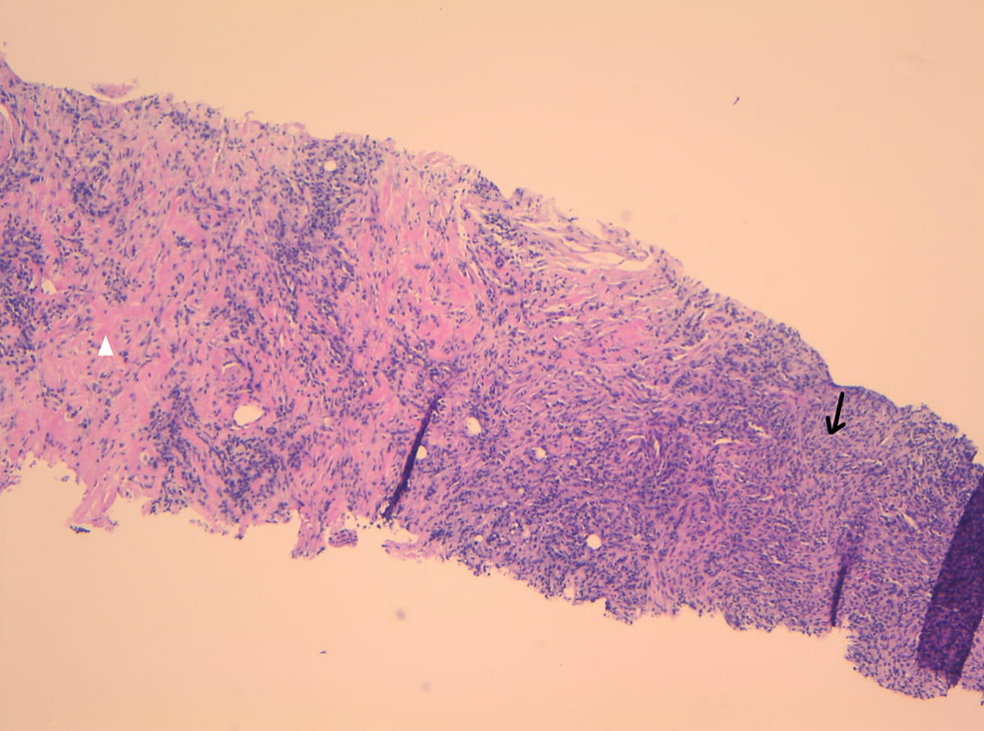
Inflamed tissue (black arrow) with pink areas of storiform fibrosis (white arrowhead) - H&E 5X ocular H&E: hematoxylin and eosin

**Figure 3:**
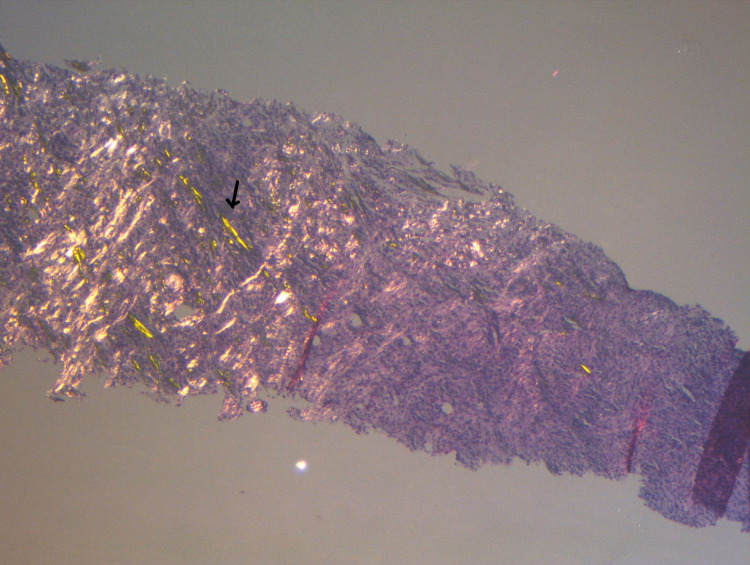
Polarized light shows intense scarring (black arrow) - H&E 5X ocular H&E: hematoxylin and eosin

Core needle biopsy showing storiform fibrosis and abundant plasma cells is presented in Figure 2, and a slide showing polarized light highlighting intense scarring is depicted in Figure 3.

**Figure 4:**
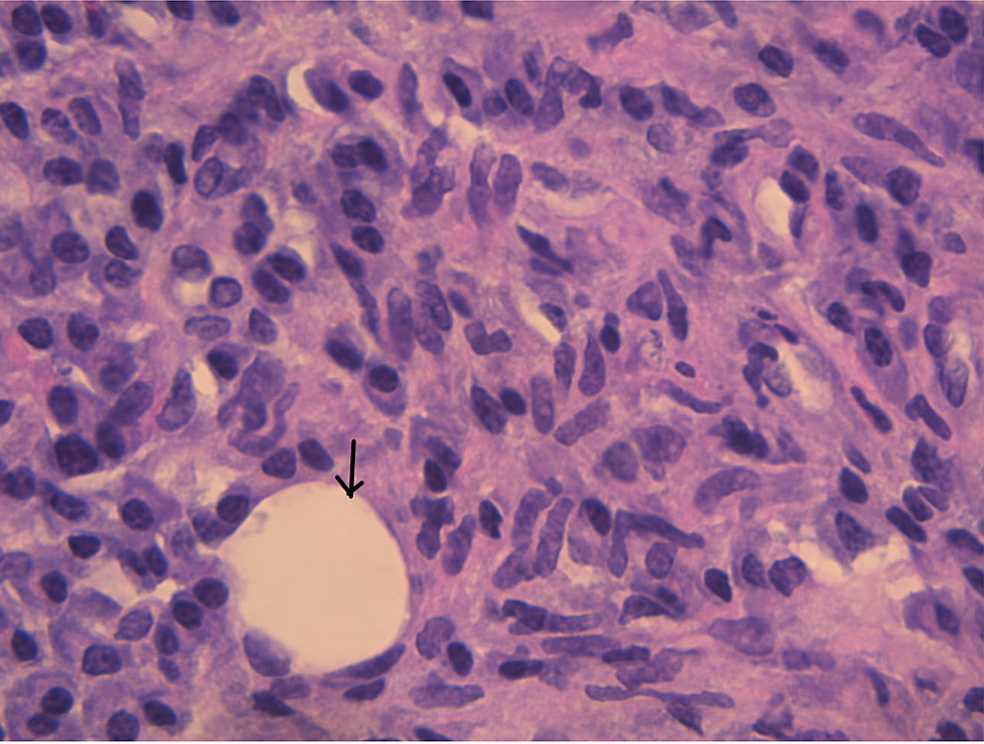
Only one adipocyte (black arrow) remains in this field flooded by macrophages, plasma cells, and occasional lymphocytes - H&E 63X ocular H&E: hematoxylin and eosin

The patient later underwent a CT-guided transgluteal biopsy of the retroperitoneal mass, which was highly suggestive of IgG4-related inflammation with microscopic evaluation showing scarring and fibrosis, which is occasionally storiform and obliterates fat, with lymphocytes, eosinophils, and plasma cells (Figure 4).

**Figure 5:**
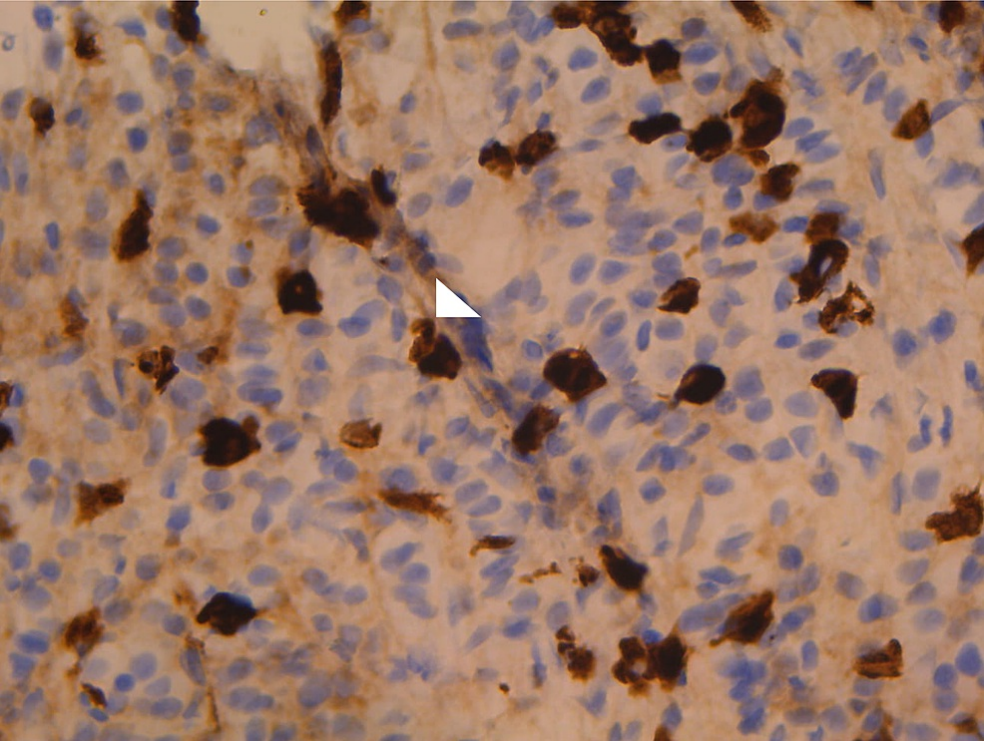
45 diagnostic IgG4-positive plasma cells - IgG4 immunostain 400X ocular The image captures about half the field visible under the microscope IgG4: immunoglobulin G4

The following image constitutes the most important immune slide, which shows 45 IgG4-positive plasma cells noted under a 400X field (Figure 5).

**Figure 6:**
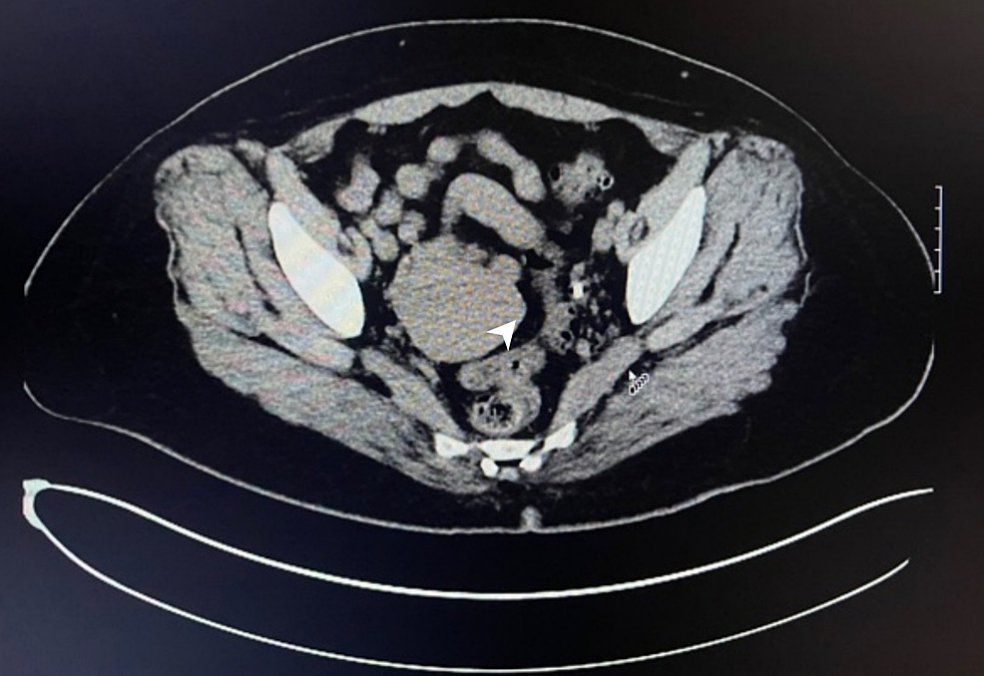
CT abdomen and pelvis after treatment showing resolution of mass (location indicated by a white arrowhead) CT: computed tomography

There was no evidence of sarcomatoid carcinoma (negative CK AE1/AE2 stain), granulomas, or vasculitis. Serum IgG4 level was measured and found elevated at 250 mg/dL. The patient was started on broad-spectrum antibiotics for possible PID/UTI. Urology was consulted, after which a left percutaneous nephrostomy tube and left ureteral stent were placed. The patient was referred to our rheumatology clinic, after which she was started on prednisone 10 mg oral daily. One month later, she was started on azathioprine (AZT) 75 mg twice daily and received RTX 1 g IV in two doses, two weeks apart. On follow-up, the patient was found to have developed gastrointestinal symptoms likely as a side effect from AZT, which was then discontinued. She was started on methotrexate 12.5 mg subcutaneous once weekly along with daily oral folic acid. Repeat CT scan of the abdomen and pelvis about seven months after the treatment showed complete resolution of the retroperitoneal mass (Figure 6).

## Discussion

IgG4-RD is an immune-mediated fibroinflammatory condition that can affect multiple organs [5,6], especially the lymph nodes, pancreas, salivary glands, kidneys, bile ducts, and lacrimal glands. Common forms of disease manifestation include type I autoimmune pancreatitis, IgG4-related sclerosing cholangitis, and RPF. IgG4-RD was originally described in Japan, with 74-86.6% of all reported cases being Japanese [7]. However, it was likely underreported in the western world earlier, with IgG4-RD now described in nearly all ethnic groups across all continents. A recent review of cases at a North American institution showed 80% to be of Caucasian ethnicity [8]. Hamano et al. first described the association between autoimmune (sclerosing) pancreatitis and elevated serum IgG4 in 2001 [9]. However, it was in 2003 that the systemic nature of IgG4-RD was discovered when patients with autoimmune pancreatitis were also observed to have extrapancreatic manifestations [10], and that was when the basics of this condition began to be constructed.

The hallmarks of IgG4-RD are dense lymphoplasmacytic infiltrations with a predominance of IgG4-positive plasma cells in the affected tissue, usually accompanied by some degree of fibrosis that has a characteristic "storiform" pattern (represented by a cartwheel appearance of the fibroblasts and inflammatory cells) and often by obliterative phlebitis and increased eosinophils [11]. Serum IgG4 levels are elevated (defined as >135 mg/dL, >121 mg/dL, or >86 mg/dL, depending upon the laboratory) in approximately two-thirds of the patients [12]. Although elevated serum IgG4 levels are not always specific for IgG4-RD, the elevation of IgG4 levels often correlates with the activity of the disease and the number of organs involved. RPF (previously described as chronic periaortitis) is a rare collagen vascular disorder that is characterized by the deposition of fibroinflammatory tissue and development of extensive fibrosis around the abdominal aorta and the iliac arteries, which often spreads within the retroperitoneal space and involves surrounding structures, notably the ureters, leading to entrapment and obstruction. The disease was first described by French urologist Joaquin Albarran in 1905. However, it was John Ormond who contributed to identifying RPF as an independent clinical entity, with his hypothesis from 1948 claiming that bilateral ureteral obstruction was caused by retroperitoneal inflammation [13]. Therefore, RPF is also known as Ormond’s disease.

Most (>70%) cases of RPF are thought to be idiopathic [14], and the remainder may be secondary to various causes such as malignancies, drugs, infections, injuries, radiotherapy, or surgery [15]. Idiopathic RPF is one of three manifestations of chronic periaortitis (CP), the others being perianeurysmal RPF and inflammatory abdominal aortic aneurysms. Retroperitoneal involvement was reported in 20-56% of patients with IgG4-RD, and IgG4-RD is responsible for 57-59% of idiopathic RPF [16]. RPF associated with IgG4-RD shows bilateral hydronephrosis in 25% of reported cases and unilateral hydronephrosis in 75% of cases [1].

RPF develops insidiously as the initial symptoms are nonspecific. Most common symptoms include low-back, flank, or abdominal pain, often radiating to the groin and/or side of the thigh. The pain is usually dull and persistent, with no decrease at rest. Initially, non-steroidal anti-inflammatory drugs can relieve the pain, but this effect is usually temporary. In the case of involvement of the ureters, the pain can have a colic character. Patients often complain of constipation, and occasionally the involvement of the duodenum results in obstructive symptoms. Less frequently reported symptoms include scrotal swelling, hydrocele, varicocele, testicular pain and swelling, and deep vein thrombosis of the lower limbs, which are related to compression of the retroperitoneally located lymphatic vessels and veins. Entrapment of arteries can result in renovascular hypertension, intermittent claudication, or intestinal ischemia. Patients may also have constitutional symptoms such as fever, fatigue, muscle and joint pain, weight loss, and loss of appetite. Physical examination is nonspecific. In some cases, lumbar or abdominal tenderness is present, and a fibrous mass can be felt through the abdominal wall in very rare instances. The nonspecific clinical picture often significantly prolongs the time between the onset of symptoms and the correct diagnosis, leading to complications related to the advanced fibrotic process. Hydronephrosis, related to ureteral obstruction and found in 47-100% of patients, is the most frequent and the most severe form of complication. In over 50% of cases, it has a bilateral character. The frequency of renal involvement is 7-24.6% [1,2]. Tubulointerstitial nephritis is the most common type of IgG4-related renal disease. Other types include glomerular diseases, such as membranous glomerulonephritis, and renal pelvic lesions, like IgG4-related pyelitis. Without glomerular involvement, renal disease leads to very mild or no symptoms in most patients. It usually presents with mild proteinuria with or without hematuria.

Diagnosis of IgG4-RD is based on the combination of characteristic clinical, serologic, radiologic, and histopathologic findings. Features include mass-like swelling of involved organs, a lymphoplasmacytic infiltrate with predominant IgG4-positive plasma cells, and a variable degree of fibrosis that has a characteristic storiform pattern. The presence of these findings, often together with mild tissue eosinophilia, is strongly suggestive if accompanied by increased numbers of IgG4-positive plasma cells in the affected tissue. Depending on the type of tissues involved, the number of IgG4-positive plasma cells per high-power field (HPF) consistent with or suggestive of IgG4-RD varies. Tissue IgG4-positive cell counts and the ratios of IgG4- to total IgG-positive cells are considered secondary in importance to the histopathologic appearance of the tissue. Generally, the minimum value required for establishing the diagnosis for most tissues ranges from 30 to 50 IgG4-positive cells/HPF. However, in some organs or tissues, including the kidney and others, 10 IgG4-positive plasma cells/HPF may be sufficient. In addition, serum IgG4 levels are elevated in approximately two-thirds of the patients. Guidelines for diagnosis have been described by the Japanese Criteria, the American College of Rheumatology, and the International Consensus Guidance Statement on the Management and Treatment of IgG4-RD.

All patients with symptomatic, active IgG4-RD require treatment. Some patients require urgent treatment, such as those with the disease in the pancreas, biliary tree, aorta, mediastinum, kidneys, retroperitoneum, and mesentery. Urgency is needed because uncontrolled disease in these organs can lead to irreversible damage. A subset of patients with asymptomatic IgG4-RD also requires treatment as a subclinical disease can lead to severe, irreversible sequelae. Some patients with asymptomatic IgG4-RD can be managed without treatment, e.g., asymptomatic lymphadenopathy or mild submandibular gland enlargement [17].

Therapy for this disease has focused on GCs to date, which appears to be highly effective in induction therapy, unless contraindicated [18]. Treatment response is usually seen within two to four weeks. Major drawbacks of GCs are the side effects as well as the need for maintenance therapy. Thus, drugs such as AZT, mycophenolate mofetil, or methotrexate have been used as GC-sparing agents. Besides, more intensive therapies such as cyclophosphamide, fludarabine, and bortezomib have been reported to be of benefit in IgG4-RD patients [19]. Some patients require the combination of GCs and a steroid-sparing immunosuppressive agent from the start of the treatment as GCs may fail to control the disease.

RTX, which is another treatment option, achieves its effects by depleting the subset of CD20-positive B cells that differentiate into the short-lived plasma cells producing the disease-associated IgG4. The treatment results with RTX in many studies strongly suggest that B-cell depletion is an effective strategy in many patients with IgG4-RD because of the strikingly targeted effect on the IgG4 subclass of IgG. Repeated treatment cycles with RTX lead to a progressive decrease in serum IgG4 concentrations, which is used as a biomarker in subsets of patients with IgG4-RD. As evidenced by our patient’s response to the treatment, B-cell depletion strategies offer a promising treatment approach toward IgG4-RD [20]. Certain patients benefit from maintenance therapy following a successful course of induction therapy.

## Conclusions

IgG4-RD is an immune-mediated fibroinflammatory condition that can affect multiple organs. RPF is one form of its manifestations, which develops insidiously because of nonspecific initial symptoms. Symptoms of IgG4-related RPF (IgG4-RRF) include low-back, flank, or abdominal pain. IgG4-RRF shows bilateral hydronephrosis in 25% of reported cases and unilateral hydronephrosis in 75% of cases. Diagnosis involves multiple criteria and MRI might be superior to CT for the characterization of findings. RTX may be a promising therapy to induce and maintain remission in patients with IgG4-RRF.
